# Cetacean Host-Pathogen Interaction(s): Critical Knowledge Gaps

**DOI:** 10.3389/fimmu.2018.02815

**Published:** 2018-11-28

**Authors:** Giovanni Di Guardo, Cinzia Centelleghe, Sandro Mazzariol

**Affiliations:** ^1^Faculty of Veterinary Medicine, University of Teramo, Teramo, Italy; ^2^Department of Comparative Biomedicine and Food Science, University of Padua, Padova, Italy

**Keywords:** cetaceans, *Cetacean Morbillivirus*, *Herpesvirus*, *Brucella ceti*, *Toxoplasma gondii*, host-pathogen interaction, cell receptors, immunotoxic pollutants

Within the broad range of viral and non-viral pathogens infecting cetaceans, *Cetacean Morbillivirus* (CeMV), *Herpesvirus* (HV), *Brucella ceti*, and *Toxoplasma gondii* are of special concern, due to their impact(s) on the health and conservation of free-ranging cetacean populations worldwide (1). The most “paradigmatic” example in this direction is represented by CeMV, which throughout the last 3 decades has caused more than 10 mass mortality outbreaks among different cetacean species and populations across the globe (2, 3).

Cetaceans live permanently in the marine environment, a peculiar feature differentiating them from pinnipeds, that are also susceptible to morbilliviral infections. This has been clearly shown, for instance, by the dramatic *Phocine*/*Phocid Distemper Virus* (PDV) and *Canine Distemper Virus* (CDV) epidemics among North Sea common seals (*Phoca vitulina*) and Lake Bajkal seals (*Pusa siberica*) as well as Caspian seals (*Pusa caspica*), respectively (4). Due to their “in-water-only” life, stranded cetaceans play a key role as “sentinels” (potentially) able to “recapitulate” the “natural history, evolution, ecology, epidemiology, and encounters” of infectious *noxae*, on one side, and cetacean hosts, on the other. Consequently, based upon their crucial relevance as “health and conservation biomonitors” for their increasingly threatened “conspecifics and heterospecifics” living in the open sea, a detailed *post-mortem* examination of stranded cetaceans on behalf of specifically trained veterinarians is mandatory!

These concepts are nicely exemplified by the *motto* “*Hic est locus ubi mors gaudet succurrere vitae*.” This phrase, written in the nineteenth century by Luciano Armanni—the co-discoverer of “*Armanni-Ebstein Diabetic Nephropathy*” (5) -, stands at the entrance of the autopsy room of “Ospedale degli Incurabili” in Naples, Italy. Literally translated, it means “*This is the place where death is pleased to support life*.”

Taking into consideration all the above, we believe there are still a number of critical “knowledge gaps” regarding “cetacean host(s)-pathogen(s) interaction(s),” with special emphasis on the 4 herein dealt infectious *noxae* and, most likely, also in relation to any other pathogen infecting wild cetaceans.

These “knowledge-deficient areas” may be identified as follows: (1) characterization of the cell receptor(s) allowing infection; (2) interaction(s) and effects of chemical pollutants on the expression levels of the aforementioned cell receptors; (3) pathogenetic evolution of the concerned infections in T helper 1 (Th1)-dominant versus (vs.) Th2-dominant cetacean individuals; (4) effects of pregnancy-associated immune *status* on the infectious potential of the herein dealt pathogens; (5) usefulness of cetaceans and their pathogens as models for human disease.

The present Opinion Article, after a brief introduction on these 5 issues, will critically address each of the aforementioned knowledge gaps.

As far as issue (1) is specifically concerned, CeMV, HV, *B. ceti*, and *T. gondii* may infect several cetacean hosts, with the latter 2 agents also carrying a zoonotic potential (1). Since different tissues from susceptible cetaceans may be colonized by the 4 herein dealt pathogens (1, 2), a detailed characterization of the cell receptor(s) allowing their entry into (and subsequent shedding from) host's tissues would be of paramount relevance. In this respect, as with other animal and human morbilliviruses, the lymphotropic behavior typically displayed by CeMV is specified by the lymphoid cell receptor “*Signaling Lymphocyte Activation Molecule*” (SLAM/CD150) (2). Notwithstanding the above, the cell receptor(s) targeted by this lympho-epithelio-neurotropic virus within the central nervous system (CNS) from susceptible cetacean hosts is/are still unknown (6, 7).

Noteworthy, striped dolphins (*Stenella coeruleoalba*) infected by *Dolphin Morbillivirus* (DMV, a CeMV strain) may occasionally develop a peculiar, “brain-only” form of infection sharing neuropathologic similarities with “*Subacute Sclerosing Panencephalitis*” (SSPE) and “*Old Dog Encephalitis*” (ODE) in *Measles Virus* (MeV)-infected humans and CDV-infected canines, respectively (2, 8–10). Despite the recent characterization of the neuronal and non-neuronal cell populations residing in the brain from striped dolphins affected by such neuropathy (11), the receptor(s) allowing viral persistence and spread within their brains—as well as in those from SSPE-afflicted patients and ODE-affected dogs—is/are also undetermined (6, 7, 12).

Identical considerations apply to HV, *B. ceti*, and *T. gondii*, provided that the cell receptor(s) allowing their entry into and subsequent dissemination throughout cetacean hosts' tissues have not been yet identified, to the best of our knowledge. Making specific reference to *B. ceti*, the host's cellular prion protein (PrP^C^) has been recently hypothesized to serve as a neuronal cell receptor for this zoonotic microorganism causing fatal neurobrucellosis in striped dolphins (13). Indeed, PrP^C^ had been previously recognized as a receptor for *B. abortus* heat shock protein (HSP)60—a member of the GroEL family of chaperonins—on murine macrophages, thereby allowing bacterial internalization and establishment of *B. abortus* infection inside these cells (14).

As far as concerns issue (2), it is well established that a wide range of persistent environmental pollutants may heavily accumulate in cetacean tissues, especially in those of “top predators” like dolphins and other Odontocetes, with simultaneous “biomagnification” processes additionally occurring in most cases (15). Many of these pollutants, as in the case of lipophilic polychlorinated biphenyls (PCBs), dioxins and dioxin-like substances, along with methylmercury (MeHg), have also been shown to exert powerful immunotoxic effects (16). Notwithstanding the above, we are not aware of any study investigating the relationship(s), if any, between pollutant-related immunotoxicity, on one side, and the tissue expression profiles, on the other, of given cell receptors (e.g., SLAM/CD150) for highly immunosuppressive agents like CeMV (6). This should be regarded as another critical knowledge gap within the general framework of “cetacean host(s)-pathogen(s) interaction dynamics.” Within such context, the growing concerns over the exponentially increasing plastic pollution of oceans and seas across the entire globe should be also taken into account. As a matter of fact, relevant health- and conservation-related issues arise for fish, birds and aquatic mammals, due to their prolonged exposure to micro-nanoplastics through the marine food web(s). Furthermore, the documented roles of “plastic debris” as an “attractor and concentrator” for many persistent pollutants like PCBs, dioxins and other organochlorine (OC) and non-OC compounds (15), as well as for a huge number of invertebrate organisms (17), would deserve special consideration. In this respect, plastics/micro-nanoplastics-based “rafts” have been recently hypothesized to play a role also in the ecology and epidemiology of *T. gondii* infection (18). This could be of interest, provided that the Scientific Community has not yet clarified by which modalities and dynamics striped dolphins and other typically “pelagic” or “offshore” cetaceans may acquire an “oro-faecally transmitted infection” characterized by a “land-to-sea flow,” as in the case of that caused by the zoonotic protozoan *T. gondii* (19).

As far as issue (3) is specifically concerned, among the many lessons provided by natural history of *Human Immunodeficiency Virus* (HIV) infection in mankind, we have learned that Th2-dominant patients are much more prone than their Th1-dominant “counterparts” to develop “full-blown AIDS” (“*Acquired Immunodeficiency Syndrome*”) in the time course of HIV infection (20). In this respect, while in recent years we have also learned quite a bit on the pathogenetic evolution of other human and animal viral and non-viral infections in Th1-dominant vs. Th2-dominant individuals, we are unaware, on the contrary, of any published reports dealing with the pathogenetic behavior of CeMV infection—as well as of HV, *T. gondii*, and *B. ceti* infections—among Th1-dominant vs. Th2-dominant cetacean hosts.

This could of relevance also in relation to issue (4), given that a reduced efficiency of host's immune response is physiologically observed during pregnancy (21). Indeed, several cases of DMV infection have been recently described both in newborns and in cetacean fetuses (6, 22–24). This concern and its related knowledge gap are additionally amplified by the documented occurrence of cases of DMV infection in cetacean species—mainly from the Western Mediterranean Sea—into which the virus has apparently “jumped,” most likely as the result of recent “spillovers” from DMV-infected striped dolphins (22–24).

Finally, with specific reference to the last of the 5 herein dealt issues, the potential role of cetaceans as “*models for human disease*” should be also taken into account (25). In fact, as recently reported for Alzheimer's disease-related neuropathology in striped and bottlenose dolphins (*Tursiops truncatus*) (26, 27), and as previously described for the aforementioned “brain-only” forms of DMV infection among striped dolphins (6, 8–10), cetaceans and—more in general—aquatic mammals could serve as valuable “models” also in “*Comparative Immunology and Immunopathology*.”

This could apply, in parallel with the herein dealt “host(s)-pathogen(s) interaction dynamics,” also to the “ontogeny” and “evolutionary phylogeny” of cetaceans' immune response as well as to further key issues like “immune tolerance,” “autoimmunity,” and “immune surveillance against neoplasia,” just to cite a few.

In conclusion, deepening our understanding of “host(s)-pathogen(s) interaction(s)” in cetaceans and, more broadly, in marine mammals, may provide not only a very useful and insightful “set of tools” to monitor and protect their increasingly threatened health and conservation, but also a reliable and precious source of knowledge highlighting the simultaneous role of cetaceans as putative “*models for human (and animal) disease*” as well as for “*Comparative Immunology and Immunopathology*.”

## Author contributions

GDG wrote the first draft as well as the first revision's draft of the present Opinion Article, to be included in the Research Topic *Comparative Immunology of Marine Mammals*, with CC and SM subsequently integrating and providing a thorough and critical revision of both the aforementioned manuscript's drafts.

### Conflict of interest statement

The authors declare that the research was conducted in the absence of any commercial or financial relationships that could be construed as a potential conflict of interest.
